# Effect of the Support, Educate, Empower Personalized Glaucoma Coaching Program on Medication Adherence: The SEE Program Randomized Clinical Trial

**DOI:** 10.1001/jamaophthalmol.2026.0001

**Published:** 2026-02-26

**Authors:** Paula Anne Newman-Casey, Leslie M. Niziol, Ming-Chen Lu, Deborah Darnley-Fisch, Nauman Imami, Jamie Mitchell, Chamisa MacKenzie, Michele Heisler

**Affiliations:** 1 Department of Ophthalmology and Visual Sciences, University of Michigan, Ann Arbor; 2 Department of Ophthalmology, Henry Ford Health System, Detroit, Michigan; 3 School of Social Work, University of Michigan, Ann Arbor; 4 Department of Internal Medicine, University of Michigan, Ann Arbor; 5 Department of Health Behavior and Health Equity, School of Public Health, University of Michigan, Ann Arbor

## Abstract

**Importance:**

Medication nonadherence contributes to vision loss from glaucoma, a leading cause of irreversible blindness in the US. Intervention to improve adherence can benefit health outcomes and quality of life, while reducing health system and public health burden.

**Objective:**

To compare the Support, Educate, Empower (SEE) intervention with standard written education on glaucoma medication adherence.

**Design, Setting, and Participants:**

The SEE study was a parallel, nonmasked, 1:1 randomized clinical trial testing intervention superiority. Participants were recruited from the University of Michigan (UM) and Henry Ford Health System (HFHS) between April 27, 2021, and December 18, 2023. Adults with glaucoma taking 1 or more ocular hypotensive eye drop medications who self-reported adherence of 85% or lower were eligible.

**Intervention:**

The SEE intervention is a 6-month motivational interviewing-based glaucoma health coaching program administered by a nonphysician counselor, including 3 in-person sessions, 4 between-visit phone calls, personalized multimedia glaucoma education, and automated medication reminders. The control consisted of usual care and 3 mailings of standard written glaucoma education.

**Main Outcomes and Measures:**

Medication adherence (primary outcome) was electronically monitored (AdhereTech) and calculated as the percentage of doses taken on time divided by those prescribed over the 6-month study period. Change in glaucoma-related distress (secondary outcome) was also investigated.

**Results:**

Of 236 participants enrolled (108 in UM and 128 in HFHS), 235 (mean [SD] age, 67.3 [10.9] years; 124 [53%] female) were randomized to the SEE intervention (n = 117) or control (n = 118). Participant characteristics were balanced between groups. Mean (SD) self-reported adherence was 63.9% (17.9%). Medication adherence was significantly better in the SEE intervention group compared with that among control participants (mean [SD], 77.6% [19.7%]; n = 113 vs 58.0% [25.2%]; n = 114; difference, 19.7%; 95% CI, 13.7 to 25.6; *P* < .001), and more achieved 80% or greater adherence (62 of 113 [54.9%] vs 27 of 114 [23.7%]; *P* < .001). The difference in change in glaucoma-related distress between intervention and control was −0.3 (95% CI, −0.5 to −0.1) after adjusting for baseline distress.

**Conclusions and Relevance:**

The SEE glaucoma coaching program improved glaucoma medication adherence and reduced glaucoma-related distress compared with standard written education. The SEE program represents an evidence-based method to improve medication adherence necessary to improve glaucoma outcomes.

**Trial Registration:**

ClinicalTrials.gov Identifier: NCT04735653

## Key Points

**Question:**

Does a personalized, glaucoma health coaching program increase medication adherence compared with standard written education in adults with glaucoma and poor self-reported adherence?

**Findings:**

In this parallel 1:1 randomized clinical trial (n = 235), participants who received the Support, Educate, Empower (SEE) intervention had clinically and significantly better medication adherence than those receiving standard written education. Glaucoma-related distress also decreased more in intervention participants.

**Meaning:**

This personalized intervention improved glaucoma medication adherence and glaucoma-related distress more than standard written education, which may confer both improvements in disease control and in quality of life.

## Teaser

This randomized clinical trial compares the use of the Support, Educate, Empower program with standard of care for medication adherence.

## Introduction

Improvements in treatments have enabled people to live longer with chronic diseases. Half of adults in the US live with at least 1 major chronic condition, and 27% with 2 or more chronic conditions.^1^ About 62% of medical expenditures of US adults older than 60 years are to treat chronic diseases.^2,3^ In 2012, the Institute of Medicine called for health care system improvements to better support chronic disease self-management.^4^ It recommended 9 self-managed chronic diseases, including vision loss, in which to test and implement population-based interventions.

Despite the availability of effective treatments, glaucoma remains the leading cause of irreversible vision loss and blindness among Black individuals and the second cause of irreversible blindness overall in the US. Three million Americans currently live with glaucoma, and that number is projected to rise to 7.3 million by 2050 as the population ages.^5^ Nonadherence to daily eye drop medications—the treatment for 73% of patients with glaucoma ^6^—is a key modifiable driver of vision loss in glaucoma.^7,8^ Patients with glaucoma do not use eye drops as scheduled at least 40% of the time.^9^

We designed the Support, Educate, Empower (SEE) personalized glaucoma coaching program to be a multimodal chronic disease self-management support program for people living with glaucoma who self-report poor glaucoma medication adherence.^10^ We then compared the SEE intervention to an enhanced care control (control) that added 3 mailings of standard written glaucoma education to usual care. We examined the SEE program’s impact at 6 months on prespecified outcomes: (1) electronically monitored glaucoma medication adherence (primary outcome), (2) glaucoma-related distress (secondary outcome), and (3) intraocular pressure (IOP; exploratory outcome).^11^

## Methods

The SEE trial was a parallel, nonmasked, 1:1 randomized clinical trial testing intervention superiority. A full description of the study methodology has been published^11^ and is provided in Supplement 1. Briefly, adults with glaucoma taking 1 or more ocular hypotensive eye drop medications who spoke English were recruited from the University of Michigan (UM; Ann Arbor, Michigan) and Henry Ford Health System (HFHS; Detroit, Michigan) ophthalmology clinics between April 27, 2021, and December 18, 2023. Potentially eligible patients were identified in electronic health records and mailed a study recruitment letter with an option to opt out of phone recruitment. Those who did not opt out verified eligibility and interest in participation by telephone. Those who self-reported adherence of 85% or lower using a validated instrument^12^ were included, as a response of 85% or lower optimized the sensitivity and specificity of electronically monitored adherence being less than 80%.^13^ Those who were eligible, agreed to participate, and provided written informed consent were enrolled. Participants were block randomized 1:1 to either treatment or control and followed for 6 months, up to July 31, 2024. Glaucoma severity was assessed by visual field testing, and medical comorbidities were assessed via the Charlson Comorbidity Index^14^ at baseline. This study was approved by the institutional review boards at both sites, is registered at ClinicalTrials.gov (NCT04735653), and followed the Consolidated Standards of Reporting Trials (CONSORT) reporting guideline.

**Figure 1.**
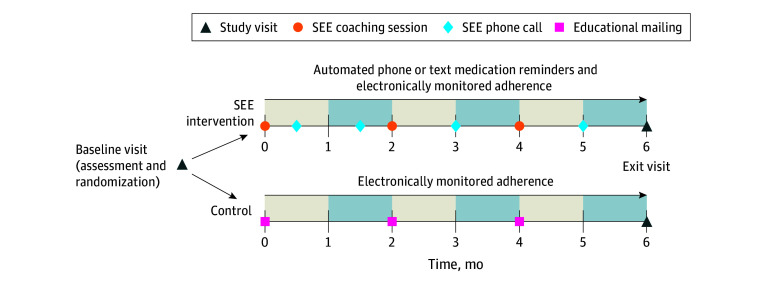
The Support, Educate, and Empower (SEE) Program Trial Schedule for SEE Intervention and Control Participants

We designed the SEE program with iterative feedback from patients with glaucoma and experts in motivational interviewing.^10^ Trained nonphysician coaches^15^ delivered the program, which included personalized multimedia glaucoma education, script-supported, motivational interviewing–based coaching,^16,17^ medication reminders (visual or audible alerts or automated text messaging or phone calls), and medication adherence monitoring. At each of the 3 in-person coaching sessions, a plan to integrate glaucoma medications into daily routines and questions for participants’ ophthalmologists were generated. At the coaching sessions and 4 between-visit phone calls (Figure 1), the coach discussed the adherence score and barriers to self-management. Fidelity to the motivational interviewing–based coaching was assessed by trainers from the Motivational Interviewing Network of Trainers,^18^ who used a standardized rubric^15,19^ to grade a random selection of 10% of encounters. The control group received mailings of educational materials from the American Academy of Ophthalmology, National Eye Institute, and Glaucoma Research Foundation every 2 months (Figure 1). All participants received usual glaucoma care from their physicians.

During the 6 months between study visits, medication-taking behavior was monitored electronically (Adheretech). Medication adherence was calculated as the percentage of doses taken on time divided by doses prescribed over the 6-month study period. Adherence was measured at the medication level (not at the eye level) using electronic monitors with a bottle-in-bottle technique where all glaucoma medications were placed inside separate electronic pill bottles. When the bottle was opened, the time and date was transmitted to our database. An adherent event was defined as using an eye drop medication within a specified time window of a dose on the previous day as specified in the protocol (Supplement 1). Changes in medications prescribed during the 6-month period were taken into account. Four calculations were performed: (1) participants were censored at the time of withdrawal, loss to follow-up, or disengagement, and times when monitors malfunctioned or were not used (eg, for hospitalization or vacation) were excluded; the remaining 3 calculations included censored times and considered the participant (2) 0% adherent during those times (worst case), (3) 100% adherent during those times (best case), or (4) 0% adherent if in the treatment group and 100% adherent if in the control group during those times (worst-best case). Adherence was assessed both as a continuous measure (0%-100% adherence) and a binary outcome (≥80% adherence vs <80% adherence). The secondary outcome, glaucoma-related distress (eTable 1 in Supplement 2), was measured at study enrollment and exit via a 17-item questionnaire adapted from the diabetes distress scale.^20^ Glaucoma-related distress was calculated as a mean of each item’s score (items scored on a Likert scale from 1 to 6, where higher scores denote more distress). The exploratory outcome, IOP, was measured in each eye 3 times consecutively at study enrollment and exit (iCare tonometer). Adverse events were recorded when identified, including death for any cause and at study-related IOP checks. When IOP was over target, patients were escorted to the glaucoma clinic for an urgent visit, and the treating clinician was notified. The study protocol and statistical analysis plan, including details of the deviations from the planned analyses that were undertaken due to the nonnormality of the adherence outcomes and differences in sample characteristics by site, are described in Supplement 1.

### Statistical Analysis

This study was designed to detect a difference in mean (SD) medication adherence of 8 (20) percentage points and a difference in mean (SD) change in glaucoma-related distress of 1.1 (2.8) between treatment and control groups, with 80% power, 2-sided α of .05, and sample size of 97 per group. Glaucoma specialists estimated that a minimally important clinical difference for glaucoma medication adherence or the point at which they would want to integrate an intervention into their clinical practice, was an improvement in a patient’s adherence of 17.7% (95% CI, 14.6-20.8)^21^; the study was powered to detect a smaller difference than this clinically important difference. Sample size estimates allowed for a dropout rate of 20%.

Descriptive statistics summarized participant characteristics, outcomes, and adverse events, overall and stratified by group. Race, ethnicity, gender, income, and education data were collected by self-report to assess the generalizability of the intervention outcomes and assess whether outcomes differed by demographic factors to inform future program development. Medication adherence was compared between groups with Wilcoxon rank sum tests, and *P* values were adjusted for multiple comparisons for the 4 different adherence calculations using Holm adjustment. Medication adherence was categorized as <80% or ≥80%, and differences in group proportions were tested with χ^2^ tests. Change in glaucoma-related distress and change in median IOP (of 3 successive measurements) from baseline to exit visit were summarized descriptively. Group differences were analyzed using linear regression, adjusting for baseline scores, as prespecified. Model results are presented with 95% CIs. The widths of the CIs have not been adjusted for multiplicity, and the intervals may not be used in place of hypothesis testing.

Data were assumed to be missing at random, and sensitivity analyses accounting for missing data were conducted using either worse-best assumptions (primary outcome) or multiple imputation (secondary and exploratory outcomes). The missing at random assumption was deemed plausible due to similar measures of baseline self-reported adherence, glaucoma-related distress, and IOP between participants who completed vs did not complete the study (eTable 2 in Supplement 2). Missing values were imputed using R multivariate imputation by chained equations (MICE) package and accounted for measures associated with the outcome and found to be different between those who completed vs did not complete the study. All analyses followed the intent-to-treat principle such that participants were analyzed as randomized for all outcomes. A *P* value <.05 was considered statistically significant for the primary outcome. SAS version 9.4 (SAS Institute) and R version 4.4.0 (R Foundation for Statistical Computing) statistical software were used for all analyses.

## Results

**Figure 2.**
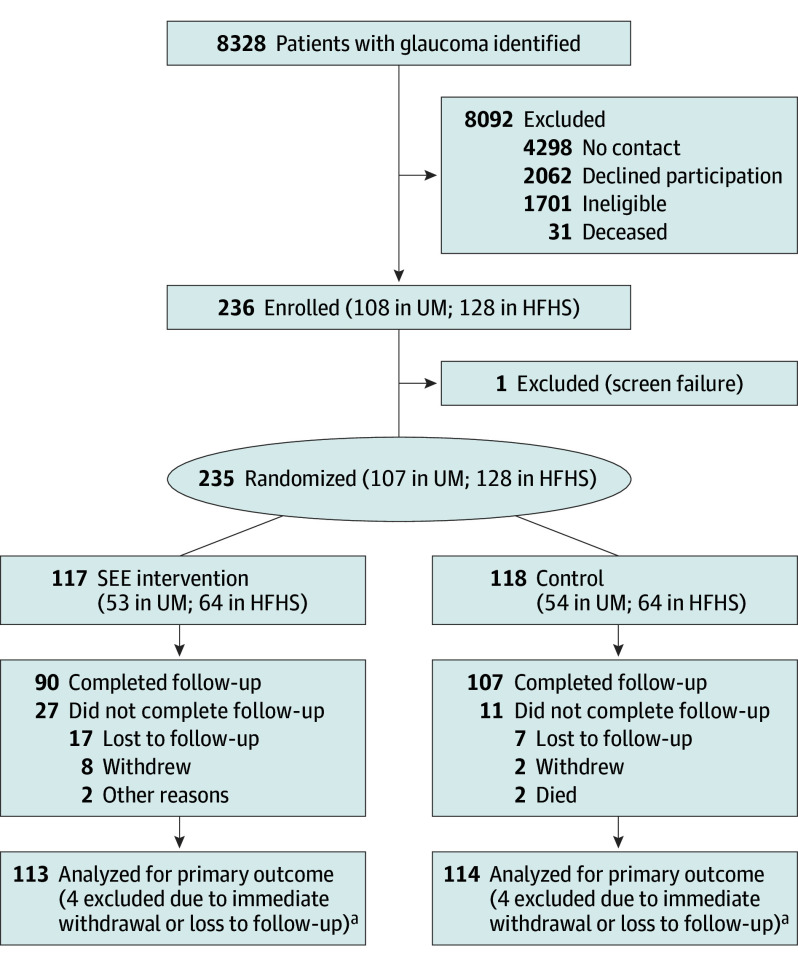
Participant Enrollment, Randomization, and Flow in the Support, Educate, and Empower (SEE) Trial HFHS indicates Henry Ford Health System; LTFU, loss to follow-up; UM, University of Michigan. ^a^Primary outcome calculated for reduced time in study for those who did not complete follow-up.

Of 236 participants enrolled, 108 were from UM (45.8%) and 128 from HFHS (54.2%), with 235 randomized to the SEE intervention (n = 117 [49.8%]) or the control (n = 118 [50.2%])( Figure 2). The mean (SD) age of participants was 67.3 (10.9) years; 124 (53%) female and 110 (47.0%) male [1 missing]; 10/227 (4.4%) Asian, 139/227 (61.2%) Black, 68/227 (30.0%) White, and 10/227 (4.4%) other, including American Indian, multiple races, and other, consolidated because of small sample sizes; and 6/208 (2.9%) were Hispanic and 202/208 (97.1%) non-Hispanic (Table 1). Nearly all participants reported having health insurance (231/233 [99.1%]) and prescription drug coverage (214/230 [93.0%]). Participants self-reported being adherent to their glaucoma medication at a mean (SD) percentage of 63.9% (17.9%) at baseline and using between 1 (89/234 [38.0%]) and 5 (1/234 [0.4%]) medications, for a mean (SD) of 3.2 (2.0) drops per day. The better glaucomatous eye of participants, as determined by mean deviation, had an average mean deviation of −4.91 (SD, 7.24) dB, IOP of 16.1 (6.8) mm Hg, and vision of 0.45 (1.37) logMAR units (Snellen equivalent, 20/56 [13.7 lines]). These characteristics were similar between treatment and control groups (Table 1). Intervention participants received a mean (SD) of 143.1 (56.1) minutes of coaching (eTable 3 in Supplement 2) and control participants, except 2 who immediately withdrew, received all 3 educational mailings.

**Table 1.** Participant Characteristics^a^

Characteristic	SEE intervention (n = 117)	Control (n = 118)
Age, y		
Total, No.	117	118
Mean (SD)	66.4 (11.5)	68.2 (10.4)
Median (IQR)	67.5 (61.2 to 72.3)	68.9 (63.2 to 73.8)
Self-reported adherence, %		
Total, No.	117	118
Mean (SD)	64.6 (16.2)	63.1 (19.6)
Median (IQR)	70.0 (50.0 to 80.0)	70.0 (50.0 to 80.0)
Better eye MD, dB^b^		
Total, No.	113	114
Mean (SD)	−4.71 (7.28)	−5.10 (7.24)
Median (IQR)	−2.27 (−6.10 to −0.65)	−2.17 (−5.42 to −0.77)
Worse eye MD, dB^b^		
Total, No.	113	114
Mean (SD)	−10.09 (9.46)	−10.47 (9.57)
Median (IQR)	−6.46 (−15.93 to −2.57)	−6.22 (−16.40 to −2.83)
Better eye IOP, mm Hg^b^		
Total, No.	113	112
Mean (SD)	16.8 (8.0)	15.4 (5.3)
Median (IQR)	15.0 (11.0 to 19.0)	15.0 (11.0 to 18.0)
Worse eye IOP, mm Hg^b^		
Total, No.	113	114
Mean (SD)	15.3 (5.3)	15.6 (6.4)
Median (IQR)	15.0 (12.0 to 18.0)	14.5 (11.0 to 18.0)
Better eye logMAR VA^b^		
Total, No.	112	114
Mean (SD)	0.44 (1.25)	0.46 (1.48)
Snellen equivalent	(20/55 [12.5 lines])	(20/58 [14.8 lines])
Median [IQR]	0.10 [0.00 to 0.30]	0.10 [0.00 to 0.18]
Snellen equivalent	(20/25 [20/20 to 20/40])	(20/25 [20/20 to 20/30])
Worse eye logMAR VA^b^		
Total, No.	112	114
Mean [SD]	0.32 [0.85]	0.28 [0.50]
Snellen equivalent	(20/42 [8.5 lines])	(20/38 [5.0 lines])
Median [IQR]	0.16 [0.00 to 0.31]	0.14 [0.00 to 0.30]
Snellen equivalent	(20/29 [20/20 to 20/41])	(20/28 [20/20 to 20/40])
No. of baseline daily doses		
Total, No.	116	118
Mean (SD)	3.1 (2.0)	3.2 (2.0)
Median (IQR)	3.0 (1.0 to 4.0)	3.0 (1.0 to 5.0)
CCI score		
Total, No.	117	118
Mean (SD)	5.2 (3.4)	5.9 (3.6)
Median (IQR)	5.0 (3.0 to 7.0)	5.0 (3.0 to 7.0)
Site, No. (%)		
UM	53 (45.3)	54 (45.8)
HFHS	64 (54.7)	64 (54.2)
Baseline medications, No. (%)		
1	44 (37.9)	45 (38.1)
2	44 (37.9)	41 (34.8)
3	22 (19.0)	24 (20.3)
4	5 (4.3)	8 (6.8)
5	1 (0.9)	0
Sex, No. (%)		
Male	53 (45.3)	57 (48.7)
Female	64 (54.7)	60 (51.3)
Ethnicity, No. (%)^c^		
Hispanic	2 (1.9)	4 (3.8)
Non-Hispanic	101 (98.1)	101 (96.2)
Race, No. (%)^c^		
Asian	6 (5.4)	4 (3.5)
Black	68 (61.3)	71 (61.2)
White	32 (28.8)	36 (31.0)
Other^d^	5 (4.5)	5 (4.3)
Education, No. (%)		
<High school	9 (7.8)	2 (1.8)
High school diploma	19 (16.5)	24 (21.1)
Some college	25 (21.7)	40 (35.1)
College degree	28 (24.4)	22 (19.3)
Graduate degree	34 (29.6)	26 (22.8)
Income, No. (%), $		
<20 000	22 (21.2)	14 (14.0)
20 000 to 40 000	28 (26.9)	26 (26.0)
41 000-60 000	20 (19.2)	18 (18.0)
61 000-80 000	7 (6.7)	12 (12.0)
81 000-100 000	7 (6.7)	9 (9.0)
101 000-120 000	8 (7.7)	6 (6.0)
>120 000	12 (11.5)	15 (15.0)
Health insurance, No. (%)	115 (99.1)	116 (99.2)
Prescription coverage, No. (%)	109 (94.8)	105 (91.3)

Abbreviations: CCI, Charlson Comorbidity Index; HFHS, Henry Ford Health System; IOP, intraocular pressure; logMAR, logarithm of minimum angle of resolution; MD, mean deviation; SEE, Support, Educate, Empower; UM, University of Michigan; VA, visual acuity.

^a^ There were no significant differences in these demographic and clinical variables between the treatment and control groups (*P* > .05).

^b^ Better and worse eye categorizations were determined by baseline MD.

^c^ Race and ethnicity data were collected via a demographic survey and reported for context and generalizability of our results

^d^ Other race groups included American Indian, multiple races, and other, consolidated because of small sample sizes.

At 6 months’ follow-up, medication adherence was significantly better among SEE intervention participants compared with control (Table 2). Adherence calculated using the censoring method showed an average difference of 19.7 percentage points (95% CI, 13.7 to 25.6; *P* < .001) between the treatment (mean [SD] of 77.6% [19.7%]; n = 113) vs control (58.0% [25.2%]; n = 114) groups. Monthly adherence during the study was stable for treatment participants while those in the control group showed decreasing adherence consistent with the Hawthorne effect (eFigure in Supplement 2). Even under the most extreme worst-best case calculation, medication adherence was an average of 11.3 percentage points higher (95% CI, 5.3 to 17.3; *P* < .001) between treatment (mean [SD], 74.2% [23.4%]; n = 117) and control (62.9% [23.6%]; n = 118). Worst-case and best-case adherence calculations also showed similar differences (Table 2). The percentage of participants achieving ≥80% adherence was greater in the intervention group than the control using the censoring method (54.9% vs 23.7%; absolute difference, 31.2%; 95% CI, 19.1 to 43.2; *P* < .001; relative risk, 2.3; 95% CI, 1.6 to 3.4; *P* < .001). This was true for all 4 adherence calculations, including for the worse-best case calculation (Table 2).

**Table 2.** Comparison of Medication Adherence Between Support, Educate, Empower (SEE) Intervention and Control Groups

	Adherence method			
	Censored	Worst case	Best case	Worst-best case
SEE Intervention				
No.	113	117	117	117
Mean (SD)	77.6 (19.7)	74.2 (23.4)	82.4 (15.8)	74.2 (23.4)
Median	82.1	78.7	87.4	78.7
No./Total No. (%) ≥80% Adherent	62/113 (54.9)	56/117 (47.9)	71/117 (60.7)	56/117 (47.9)
Control				
No.	114	118	118	118
Mean (SD)	58.0 (25.2)	54.8 (27.7)	62.9 (23.6)	62.9 (23.6)
Median	60.5	59.9	65.7	65.7
No./Total No. (%) ≥80% Adherent	27/114 (23.7)	27/118 (22.9)	32/118 (27.1)	32/118 (27.1)
Difference, mean (95% CI)^a^	19.7 (13.7-25.6)	19.3 (12.7-25.9)	19.6 (14.4-24.7)	11.3 (5.3-17.3)
Holm-adjusted *P* Value^b^	<.001	<.001	<.001	<.001
Difference, % (95% CI)^c^	31.2 (18.1-43.0)	25.0 (12.0-36.7)	33.6 (20.8-45.2)	20.7 (7.7-32.7)
Holm-adjusted *P* value^b^	<.001	<.001	<.001	.001

^a^ Difference between SEE intervention and control on mean percentage adherence.

^b^ Wilcoxon rank-sum tests for continuous measures of medication adherence and Chi-square tests for categorical measures of medication adherence. Minimally important clinical difference in glaucoma medication adherence is 17.7%.^21^

^c^ Difference between SEE intervention and control on percentage adherence ≥80%

SEE intervention participants had a greater decrease in glaucoma-related distress from baseline to exit visit compared with control participants, including a decrease in the composite score, the emotional burden subscale, and the regimen-related distress subscale. For the composite score, intervention participants (n = 90 with both surveys) had a mean (SD) baseline glaucoma-related distress score of 2.5 (0.9), which decreased to 1.9 (0.8) at study exit, compared with control participants (n = 107) whose baseline score was 2.4 (0.8) and decreased to 2.2 (0.9) at study exit. After adjustment for baseline distress, the estimated difference in change in composite glaucoma-related distress was −0.3 (95% CI, −0.5 to −0.1) between those in the intervention (−0.6, 95% CI, −0.7 to −0.4) vs control (−0.2, 95% CI, −0.4 to −0.1) (Table 3). This smaller difference (0.3) than anticipated by power calculation (1.1) was detected because the standard deviation was much smaller (0.8) than expected (2.8). Sensitivity analysis with multiple imputation showed similar results (Table 3).

**Table 3.** Changes in Glaucoma-Related Distress (Secondary Outcome) and Intraocular Pressure (IOP; Exploratory Outcome) Between Support, Educate, Empower (SEE) Intervention and Control Groups

	Estimate (95% CI)		SEE intervention vs control, estimated treatment difference (95% CI)^a^	
	SEE intervention	Control	Complete case	Sensitivity analysis
Change in glaucoma-related distress				
Composite	−0.6 (−0.7 to −0.4)	−0.2 (−0.4 to −0.1)	−0.3 (−0.5 to −0.1)	−0.3 (−0.5 to −0.1)
Emotional burden	−0.5 (−0.7 to −0.3)	−0.2 (−0.3 to 0.0)	−0.3 (−0.6 to −0.1)	−0.3 (−0.6 to −0.1)
Physician-related distress	−0.2 (−0.3 to 0.0)	0.0 (−0.2 to 0.1)	−0.2 (−0.4 to 0.1)	−0.2 (−0.4 to 0.1)
Regimen-related distress	−1.1 (−1.3 to −0.9)	−0.5 (−0.7 to −0.4)	−0.6 (−0.8 to −0.3)	−0.5 (−0.8 to −0.3)
Interpersonal distress	−0.3 (−0.5 to −0.1)	−0.2 (−0.4 to 0.0)	−0.1 (−0.4 to 0.2)	−0.1 (−0.4 to 0.2)
Change in IOP				
Median IOP, worse eye	−0.03 (−1.07 to 1.01)	−0.18 (−1.14 to 0.78)	0.15 (−1.27 to 1.56)	0.32 (−1.07 to 1.71)
Median IOP, better eye	−0.84 (−1.56 to −0.11)	−0.38 (−1.06 to 0.30)	−0.46 (−1.45 to 0.54)	−0.44 (−1.41 to 0.53)

^a^ Treatment differences for change in glaucoma-related distress and change in IOP were estimated using linear regression, adjusting for baseline scores. The complete case analysis was conducted without multiple imputation, and the sensitivity analysis was conducted with multiple imputation.

Change in IOP from baseline to exit visit did not differ between intervention and control groups in the worse eye (estimated difference: 0.15 mm Hg; 95% CI, −1.27 to 1.56) or the better eye (estimated difference: −0.46 mm Hg; 95% CI, −1.45 to 0.54) after adjustment for baseline IOP (Table 3). The sensitivity analysis with multiple imputation showed similar results (Table 3). After adjusting for baseline IOP, intervention group participants had a reduction in IOP from baseline in their better eye (n = 88; −0.84 mm Hg; 95% CI, −1.56 to −0.11), while control group participants did not (n = 102; −0.38 mm Hg; 95% CI, −1.06 to 0.30) with no change in the worse eye in either group. Descriptive summaries of glaucoma-related distress and IOP for intervention and control groups can be found in eTable 4 in Supplement 2.

To assess counselor fidelity to motivational interviewing–based coaching, audio transcripts from 118 encounters with 67 participants were assessed and scored by motivational interviewing trainers (62 from counselor 1, 56 from counselor 2). The mean (SD) motivational interviewing skill score was 5.7 (0.6) (counselor 1: 5.7 [0.7]; counselor 2: 5.7 [0.5]), which was above the competence level threshold score of 5 (*P* < .001).

Adverse events were noted in 26 participants (11.1%). High IOP was documented 32 times among 25 participants (18/117 intervention participants [15.4%]; 7/118 control participants [5.9%]; *P* = .04) (eTable 5 in Supplement 2). Death was documented for 2 control participants with cause unrelated to the study. Protocol deviations were documented for 6 participants and did not differ between groups (eTable 5 in Supplement 2).

## Discussion

In this randomized clinical trial of a diverse sample of adults with glaucoma and poor self-reported medication adherence at baseline, intervention participants achieved a statistically and clinically significant improvement of 19.7% in medication adherence compared with control participants. This adherence level exceeds the minimally important clinical difference of 17.7%.^21^ The finding was robust even to the worst-best case scenario analysis, where adherence was still 11.3% higher in the intervention group compared with the control group.

As important, SEE program participants reported significant decreases in glaucoma-related distress compared with participants in the control arm, decreases driven by decreases in emotional burden and regimen-related distress. The difference observed in treatment participants (−0.6) exceeded the minimal clinically important difference for change in glaucoma-related distress (0.25).^22^ Scores of <2.0 relate to little to no distress, 2.0-2.9 to moderate distress, and ≥3.0 to high distress.^23^ SEE coaching program participants started at moderate distress and decreased to little or no distress after program participation, while there was little change in distress level for control participants, who maintained moderate distress. Our exploratory outcome was IOP, and there was no difference in IOP between intervention and control arms.^24^ As IOP varies by an average of 6 mm Hg throughout the day^25^; as we had only a single time point measurement of IOP at baseline and exit, we were likely underpowered to detect a difference in IOP.

A network meta-analysis^26^ of randomized trials evaluating interventions to improve glaucoma medication adherence found that interventions that were more complex with multiple components tailored to patients’ unique needs were most effective. Of the 19 trials included, 4 followed adherence of 6 months or longer to mitigate the Hawthorne effect and measured adherence with electronic monitors to assess adherence more precisely than prescription refill rates, which is the protocol we followed in our trial. Of these 4 trials, 2 demonstrated an impact on adherence^9,27^ while 2 did not.^28,29^ The 2 effective interventions focused on patients with poor glaucoma medication adherence at baseline, similar to our SEE study. The 2 without effect included all patients. It makes sense to focus additional self-management support resources on patients who have poor self-reported adherence. One of the successful programs used an adherence-dependent rebate program and demonstrated a 12.2% improvement in adherence compared with control.^27^ The second, the Medication Adherence in Glaucoma to Improve Care (MAGIC) trial, demonstrated a 23% improvement in glaucoma medication adherence compared with control using a personally tailored multimedia education session that also taught eye drop instillation, followed by 6 months of a dose reminder system.^9^ The MAGIC intervention had a larger impact on medication adherence than the rebate program, with an effect size similar to the SEE study.

The SEE program adds scalability to this multifaceted approach, as the web-based program generates more than 287 million unique combinations of educational messages based on a patient’s type of glaucoma, test results, physician recommendations, barriers to glaucoma medication adherence, and support system. Therefore, the tailored education does not depend on the knowledge bank of the health educator as it did in the MAGIC trial. The SEE program also includes a glaucoma-specific motivational interviewing training program and embedded motivational interviewing–based prompts to guide the conversation between the health educator and the patient. Thus, the glaucoma coach does not need to be a trained health care professional (such as an ophthalmic technician or pharmacist).

### Strengths and Limitations

This study has multiple strengths. It was adequately powered to assess a clinically meaningful change in glaucoma medication adherence. It had a clinically relevant control group whose participants received the same information as the intervention group but without the motivational interviewing–based health coaching and adherence feedback. Electronic monitoring of glaucoma medication adherence has higher precision than either self-reported medication adherence or assessment of pharmacy refill data.^30^ Medication adherence was assessed over the 6-month program, a long enough time to mitigate the known approximately 2-month impact^17^ of the Hawthorne effect on glaucoma medication-taking behavior.

The study also has limitations. Loss to follow-up was higher in the intervention group (23%) than in the control group (9%), suggesting potential time burden of participating in the coaching program. The demographic characteristics of the participants at each of the 2 sites were different in terms of race, educational attainment, and income level. We did not adjust for site in the main outcome analysis, as we are planning a more detailed assessment of moderators of treatment effect, including site, income, race, and gender. The study included 99% of participants with health insurance, limiting generalizability to uninsured patients. This study was not designed to disentangle the effects of the various components of the SEE program. Additionally, adverse effects, such as loss of privacy or autonomy from having medication taking behavior monitored, were not measured.^31^

## Conclusions

In conclusion, the SEE personalized glaucoma coaching program demonstrated clinically significant improvements in glaucoma medication adherence and reductions in glaucoma-related distress compared with the control group receiving standard written education. To enhance scalability, the SEE Program was created with a web-based tool that creates personalized glaucoma education, a script to guide motivational interviewing-based coaching sessions, a glaucoma-specific motivational interviewing-based training program for health educators, and a rubric to grade fidelity to the intervention. We now plan to evaluate the SEE Program in a pragmatic implementation trial that examines impacts on biological outcomes as well as implementation process measures.

## Notes

Supplement 1. Trial protocol

Supplement 2. eTable 1. Glaucoma Related Distress Scale eTable 2. Comparison of participant characteristics between those who did and did not complete the SEE study eTable 3. Descriptives for duration of counseling received by participants randomized to the Support, Educate, Empower (SEE) intervention, stratified by those who finished and did not finish the 6-month intervention eTable 4. Summary of glaucoma related distress and intraocular pressure at baseline and exit visits for intervention and control groups eTable 5. Protocol deviations and adverse events eFigure. The distribution of medication adherence over time during the 6 month study period, stratified by SEE counseling intervention and control groups

Supplement 3. Data sharing statement

## References

[1] Boersma P, Black LI, Ward BW. Prevalence of multiple chronic conditions among US adults, 2018. Prev Chronic Dis. 2020;17:E106. doi:10.5888/pcd17.200130 32945769 PMC7553211

[2] US Centers for Disease Control and Prevention. Fast facts: health and economic costs of chronic conditions. chronic disease. December 10, 2024. Accessed December 3, 2024. https://www.cdc.gov/chronic-disease/data-research/facts-stats/index.html

[3] Minesota Department of Health. Treated chronic disease prevalence and spending in Minnesota. December 2022. Accessed June 10, 2025. https://www.health.state.mn.us/data/economics/docs/chroncondbrief121322.pdf

[4] Committee on Living Well with Chronic Disease. Public Action to Reduce Disability and Improve Functioning and Quality of Life, Board on Population Health and Public Health Practice, Institute of Medicine. Living Well with Chronic Illness: A Call for Public Health Action. National Academies Press; 2011.

[5] Vajaranant TS, Wu S, Torres M, Varma R. A 40-year forecast of the demographic shift in primary open-angle glaucoma in the United States. Invest Ophthalmol Vis Sci. 2012;53(5):2464-2466. doi:10.1167/iovs.12-9483d 22562841

[6] Rein DB, Zhang P, Wirth KE, . The economic burden of major adult visual disorders in the United States. Arch Ophthalmol. 2006;124(12):1754-1760. doi:10.1001/archopht.124.12.1754 17159036

[7] Newman-Casey PA, Niziol LM, Gillespie BW, Janz NK, Lichter PR, Musch DC. The association between medication adherence and visual field progression in the collaborative initial glaucoma treatment study. Ophthalmology. 2020;127(4):477-483. doi:10.1016/j.ophtha.2019.10.022 31932093 PMC7093219

[8] Shu YH, Wu J, Luong T, . Topical medication adherence and visual field progression in open-angle glaucoma: analysis of a large US health care system. J Glaucoma. 2021;30(12):1047-1055. doi:10.1097/IJG.0000000000001943 34669680 PMC8635266

[9] Muir KW, Rosdahl JA, Hein AM, . Improved glaucoma medication adherence in a randomized controlled trial. Ophthalmol Glaucoma. 2022;5(1):40-46. doi:10.1016/j.ogla.2021.04.006 33892170

[10] Killeen OJ, MacKenzie C, Heisler M, Resnicow K, Lee PP, Newman-Casey PA. User-centered design of the eyeGuide: a tailored glaucoma behavior change program. J Glaucoma. 2016;25(10):815-821. doi:10.1097/IJG.0000000000000431 27096721 PMC5067955

[11] Newman-Casey PA, Resnicow K, Winter S, . The Support, Educate, Empower personalized glaucoma coaching trial design. Clin Trials. 2023;20(2):192-200. doi:10.1177/17407745221136571 36855233 PMC10023277

[12] Chang DS, Friedman DS, Frazier T, Plyler R, Boland MV. Development and validation of a predictive model for nonadherence with once-daily glaucoma medications. Ophthalmology. 2013;120(7):1396-1402. doi:10.1016/j.ophtha.2013.01.002 23541760 PMC4016983

[13] Cho J, Niziol LM, Lee PP, . Comparison of medication adherence assessment tools to identify glaucoma medication nonadherence. Ophthalmol Glaucoma. 2022;5(2):137-145. doi:10.1016/j.ogla.2021.07.012 34358735 PMC8814049

[14] Charlson ME, Pompei P, Ales KL, MacKenzie CR. A new method of classifying prognostic comorbidity in longitudinal studies: development and validation. J Chronic Dis. 1987;40(5):373-383. doi:10.1016/0021-9681(87)90171-8 3558716

[15] Newman-Casey PA, Niziol LM, Mackenzie CK, . Personalized behavior change program for glaucoma patients with poor adherence: a pilot interventional cohort study with a pre-post design. Pilot Feasibility Stud. 2018;4:128. doi:10.1186/s40814-018-0320-6 30062043 PMC6055343

[16] Hollenhorst CN, Elliott V, Heisler M, Schneider K, Resnicow K, Newman-Casey PA. Patient experience during the support, educate, empower glaucoma coaching program to improve medication adherence: a pilot study. Ophthalmol Glaucoma. 2020;3(4):238-252. doi:10.1016/j.ogla.2020.04.016 33008556 PMC7532982

[17] Newman-Casey PA, Niziol LM, Lee PP, Musch DC, Resnicow K, Heisler M. The impact of the support, educate, empower personalized glaucoma coaching pilot study on glaucoma medication adherence. Ophthalmol Glaucoma. 2020;3(4):228-237. doi:10.1016/j.ogla.2020.04.013 33012330 PMC7528849

[18] MINT. Welcome to the motivational interviewing website! Accessed June 10, 2025. https://motivationalinterviewing.org/

[19] Newman-Casey PA, Killeen O, Miller S, . A glaucoma-specific brief motivational interviewing training program for ophthalmology para-professionals: assessment of feasibility and initial patient impact. Health Commun. 2020;35(2):233-241. doi:10.1080/10410236.2018.1557357 31878800 PMC6936335

[20] Polonsky WH, Fisher L, Earles J, . Assessing psychosocial distress in diabetes: development of the diabetes distress scale. Diabetes Care. 2005;28(3):626-631. doi:10.2337/diacare.28.3.626 15735199

[21] Kolli A, Daniel-Wayman S, Newman-Casey PA. The minimal clinically important difference in glaucoma medication adherence: interviews of glaucoma experts. Ophthalmic Res. 2021;64(3):524-528. doi:10.1159/000512924 33171476 PMC9088219

[22] Banks J, Amspoker AB, Vaughan EM, Woodard L, Naik AD. Ascertainment of minimal clinically important differences in the Diabetes Distress Scale–17: a secondary analysis of a randomized clinical trial. JAMA Netw Open. 2023;6(11):e2342950. doi:10.1001/jamanetworkopen.2023.42950 37966840 PMC10652154

[23] Fisher L, Hessler DM, Polonsky WH, Mullan J. When is diabetes distress clinically meaningful?: establishing cut points for the Diabetes Distress Scale. Diabetes Care. 2012;35(2):259-264. doi:10.2337/dc11-1572 22228744 PMC3263871

[24] Musch DC, Gillespie BW, Niziol LM, Lichter PR, Varma R; CIGTS Study Group. Intraocular pressure control and long-term visual field loss in the Collaborative Initial Glaucoma Treatment Study. Ophthalmology. 2011;118(9):1766-1773. doi:10.1016/j.ophtha.2011.01.047 21600658 PMC3161134

[25] David R, Zangwill L, Briscoe D, Dagan M, Yagev R, Yassur Y. Diurnal intraocular pressure variations: an analysis of 690 diurnal curves. Br J Ophthalmol. 1992;76(5):280-283. doi:10.1136/bjo.76.5.280 1356429 PMC504256

[26] Ha A, Jang M, Shim SR, Kim CY, Chang IB, Kim YK. Interventions for glaucoma medication adherence improvement: a network meta-analysis of randomized controlled trials. Ophthalmology. 2022;129(11):1294-1304. doi:10.1016/j.ophtha.2022.06.025 36028393

[27] Bilger M, Wong TT, Lee JY, . Using adherence-contingent rebates on chronic disease treatment costs to promote medication adherence: results from a randomized controlled trial. Appl Health Econ Health Policy. 2019;17(6):841-855. doi:10.1007/s40258-019-00497-0 31317511 PMC6885505

[28] Beckers HJM, Webers CAB, Busch MJWM, Brink HM, Colen TP, Schouten JS; Dutch Adherence Study Group. Adherence improvement in Dutch glaucoma patients: a randomized controlled trial. Acta Ophthalmol. 2013;91(7):610-618. doi:10.1111/j.1755-3768.2012.02571.x 23025424

[29] Cate H, Bhattacharya D, Clark A, Fordham R, Holland R, Broadway DC. Improving adherence to glaucoma medication: a randomised controlled trial of a patient-centred intervention (the Norwich Adherence Glaucoma Study). BMC Ophthalmol. 2014;14(1):32. doi:10.1186/1471-2415-14-32 24655814 PMC3994324

[30] Muir KW, Lee PP. Glaucoma medication adherence: room for improvement in both performance and measurement. Arch Ophthalmol. 2011;129(2):243-245. doi:10.1001/archophthalmol.2010.351 21320975 PMC3721728

[31] Simpson SH, Eurich DT, Majumdar SR, . A meta-analysis of the association between adherence to drug therapy and mortality. BMJ. 2006;333(7557):15. doi:10.1136/bmj.38875.675486.55 16790458 PMC1488752

