# Supplementary material for: Effect of the Support, Educate, Empower Personalized Glaucoma Coaching Program on Medication Adherence: The SEE Program Randomized Clinical Trial
Source: JAMA Ophthalmol. 2026 Feb 26;144(4):299–306. doi: 10.1001/jamaophthalmol.2026.0001 (PMC12947086; doi:10.1001/jamaophthalmol.2026.0001)
Supplement: Supplement 1. — Trial protocol [file jamaophthalmol-e260001-s001.pdf]

Online-Only Supplement 1

Supplement to: Newman-Casey PA, Niziol LM, Lu MC, et al. The Support, Educate, Empower Personalized Glaucoma Coaching Program Effect on Medication Adherence: SEE Program Randomized Clinical Trial

Working Title: The SEE Program

HUM00188154

Protocol & Statistical Analysis Plan

BACKGROUND AND SIGNIFICANCE

Despite evidence from randomized clinical trials that medication reduces vision loss from glaucoma, it remains the second leading cause of blindness in the United States.[1] A critical barrier to preventing vision loss is that about one-half of glaucoma patients are essentially "untreated" because they do not adhere to their medications.[2,3] Ineffective self-management behaviors and poor clinical outcomes disproportionately affect the most vulnerable members of US society.[4–7] There are programs individualized to use techniques such as tailored health communication and motivational interviewing for a wide range of chronic diseases.

(HUM00112614) We developed and pilot tested the SEE program to offer motivation interviewing based-counseling and personalized education to patients who are poorly adherent to their glaucoma medications as part of their ophthalmic care.[8–10] In addition, my team developed and pilot tested a glaucoma-specific motivational interviewing training program for ophthalmic para-professional staff.[11] In the SEE program, a trained glaucoma coach uses a web-based application to deliver personalized high-quality, counseling and education.

Our overall objective is to test whether the Support, Educate, Empower: (SEE) Personalized Glaucoma Coaching Program, compared to control care by the physician with additional written education materials, improves glaucoma eye drop adherence through a randomized clinical trial among approximately 230 glaucoma patients with poor adherence at enrollment. We will recruit from clinics that serve low-income and minority populations as these populations have a higher incidence of both poor adherence and outcomes from glaucoma.

Our central hypothesis is that glaucoma patients with poor adherence who receive motivational-interviewing based counseling and personalized education from a trained non-physician glaucoma coach through the SEE Program will improve their medication adherence. In the six-month SEE Program, health educators trained as glaucoma coaches use an eHealth program as a tool to deliver personalized education (e.g. based on each person's diagnosis, test results, physician's recommendations) and motivational interviewing-based counseling to guide patients to identify their barriers to optimal adherence and brainstorm solutions. Additionally, participants can choose any or all of their preferred modalities for receiving reminders when a medication dose is missed: an alarm (light or sound), an automated phone call or text message.

OBJECTIVE

Our overall objective is to test whether the SEE Program, compared to control care, improves medication adherence through a randomized clinical trial among glaucoma patients with poor medication adherence at enrollment.

METHODS

Intervention

Compare the efficacy of the SEE Program with control care in improving glaucoma medication adherence. We will test the efficacy of the SEE Program to improve medication adherence among glaucoma patients. Our study design is a randomized controlled clinical efficacy trial with an embedded mixed methods analysis. The primary outcome, medication adherence at six months, will be assessed objectively with electronic monitoring. We will test the efficacy of the intervention on improving medication adherence behavior while gathering data that will inform future program dissemination and implementation. We will conduct a parallel, two-armed RCT, recruiting participants (n=230) with self-reported poor adherence in equal numbers from two southeastern Michigan-based health systems {University of Michigan (UM) and Henry Ford Health System (HFHS)}; that both serve low-income, minority populations. We will oversample African Americans to comprise at least 25% of our sample to ensure adequate power to assess improvements in this sub-population. We will enroll up to 160 participants at UM or HFHS. We will stop enrollment when the goal of 230 is met combining UM and HFHS.

We chose to work in two health systems to ensure adequate recruitment of patients who are poorly adherent to their medication and willing to participate.

Rationale for the Intervention

Both empowerment theory and self-determination theory form the basis for our medication adherence intervention. The World Health Organization describes empowerment as a "process through which people gain greater control over decisions and actions affecting their health."[12] Empowerment is particularly important for people who may experience a powerlessness that can come with minority status or poverty.[13–15] In order for people to best manage their eye health, empowerment theory suggests that they must be supported to develop knowledge and skills to care for their health, coping skills for managing emotions that can negatively affect self-management, and motivation to improve health.[16] Self-determination theory posits that to change a health behavior, people must feel autonomously supported and connected to their health care provider, perceive that they are competent to engage in the behavior, and be motivated to improve.[17]

The application of MI in our study is consistent with the underpinnings of both theoretical frameworks. MI counseling engages patients by discussing priorities and obstacles to facilitate intrinsic motivation— personally compelling reasons— to change health behavior. Recent Cochrane reviews and meta-analyses of medication adherence interventions found that MI-based counseling was the most successful approach to increasing adherence across many chronic diseases.[18,19] We hypothesize that using MI-based personalized glaucoma counseling and education will increase participants' motivation, competence, and satisfaction with care, thereby increasing medication adherence and decreasing glaucoma related distress.

RESEARCH DESIGN

Training:

We will hire two health educators to be trained as glaucoma coaches to provide coaching at HFHS and UM. The health educators will complete the two-day glaucoma-specific, MI training program followed by individual coaching and supervision, led by a Motivational Interviewing Network of Trainers[20] trained counselor.[11] The program teaches the five core MI skills: asking open-ended questions, affirming, reflecting, summarizing, and obtaining permission to provide information and advice.[21] These skills help soften resistance and elicit 'change talk,' through which coaches help patients talk themselves into change. The program teaches how to express empathy, which underlies the spirit of MI and promotes rapport between the coach and the patient. The program utilizes didactics and role playing using our video collection of glaucoma patients explaining their reasons for discontinuing their medications.

Health educators will conduct three practice sessions for each of the three SEE Program sessions and follow-up phone calls (12 total practice sessions). Health educators will meet with the MI trainer weekly for two months to review these practice sessions and ensure that they meet criteria for competence before counseling patients. During the intervention, all coaching sessions will be audio-recorded. The MI trainer will review a random sample of 10% of encounters and grade them for fidelity to MI using the validated One-Pass grading tool adapted for this intervention.[22] We have used this glaucoma-specific One-Pass grading tool in our pilot study with good success. The MI trainer will meet with coaches to review the recorded and graded encounters biweekly. If the trainer notes a decrease in graded MI fidelity, she will increase the level of supervision.

Recruitment:

Participant selection will occur from the UMHS and HFHS electronic health records. We will generate a list of all patients at UM and HFHS taking ≥1 ocular hypotensive medication with a diagnosis of any kind of glaucoma, suspected glaucoma or ocular hypertension who are ≥ 18 years of age. A letter will be sent to all potentially eligible participants allowing people to opt out of phone-based recruitment.

Inclusion criteria:

- ≥18 years of age
- Taking ≥1 ocular hypotensive medication with a diagnosis of any kind of glaucoma, suspected glaucoma or ocular hypertension
- Did not opt-out from recruitment letter
- Able to instill eyedrops themselves
- Have a phone, cell or landline.

Exclusion criteria:

- Do not speak English

Have a diagnose serious mental illness (defined as schizophrenia, bipolar disorder, or a major depressive episode with psychotic features)

Diagnosed cognitive impairment

Do not instill their own eye drops

Had laser or incisional glaucoma surgery within the last 3 months or scheduled during the six-month study period

Prisoners

Active uveitis or ocular infection

Participated in pilot study

Unable to attend all study visits

*We will recruit men and women equally. We will oversample African American patients to ensure at least 25% representation in the trial sample.

UM Study staff will become registered Henry Ford Health Systems volunteers. Henry Ford Health Systems staff will become registered as UM volunteers. This will allow for staff to access clinic space and electronic health records at each institution.

At UM and HFHS, research associates will call potential participants and obtain verbal consent to give a survey assessing self-reported medication adherence to determine study eligibility. Self-reported medication adherence will be measured from a single question asking "Over the past month, what percentage of your drops do you think you took correctly?" Those who self-report <85% adherence will be invited to participate in the study.[23] Self-reported medication adherence ≤85% maximized Youden's J statistic for optimization of the sensitivity/specificity of electronically monitored adherence being <80%. Research associates will schedule a baseline visit to complete the informed consent process, the baseline survey, measurement of intraocular pressure and measurement of visual acuity and visual field, if participants have not had a reliable test with the last year (HVF SITA 24-2, ≤20% fixation losses, ≤15% false positives).

<u>Participant Assessments:</u> After eligibility has been confirmed and informed consent has been obtained, prior to randomization, a research associate will measure participants' intraocular pressure (IOP), record socio-demographic characteristics, administer a baseline survey including measures of glaucoma-related distress, glaucoma knowledge, perceived competence, motivation, self-efficacy, and autonomy support from their eye care team. IOP measurements will be taken at all in-person study visits. Surveys will be administered by study staff on a tablet with the ability to enlarge the font or have the survey read out loud. Eye drop instillation will be video-recorded. Snellen visual acuity at 20 feet will be recorded on all participants. To assess glaucoma severity, the last reliable visual field (<20% fixation losses, <15% false positives, <30% false negatives)[24] within one year of the baseline study visit was pulled from the electronic health record, or when a one was not available a Humphrey Visual Field 24-2 SITA FAST will be completed at the baseline visit. Worse eye and better eye are defined by which eye has a lower mean deviation on visual field testing. Charlson Co-morbidity Index values were calculated for each participant via medical record review at baseline.[25]

Participants will be randomized to the SEE Program (experimental) or control care by the physician with additional written education materials (control). Medication adherence will be monitored electronically for all participants from baseline through the end of the 6-month program period. A baseline measure of medication adherence will be obtained by an objective method (medication possession ratios assessed from pharmacy refill data for six months prior to study participation) and subjective method (survey). After six-months, a research associate will video-record eye drop instillation, administer a survey with the same behavioral and psychosocial measures and a quantitative program evaluation, measure IOP and conduct a 15-30 minute semi-structured interview with participants in the SEE Program arm to assess their experience with the program. Additionally, we will call participants that do or did not return for the exit visit to ask if they will complete the exit surveys over the phone. All data will be entered into the REDCap database. Following completion of the program, pharmacy refill data will be collected for an additional 12 months to assess longer-term effects of the SEE Program.

<u>Randomization:</u> After baseline testing, participants will be given their randomization assignment. We will use block randomization stratified by site to allocate participants to the intervention or control groups. A randomization list using blocks of varying sizes (2, 4, and 6) will be produced in randomization software for each site. Block

randomization maintains approximate group size balance during the trial. Varying the block size lowers the likelihood of knowing the group assignment of the next eligible participant. Randomized group status will be available to the research associate by email after baseline information and consent are entered into REDCap and eligibility is confirmed.

Retention strategies: Prior to randomization, participants are shown a two-minute animated video explaining the importance of full participation for the trial to have meaningful results. Participants will have the opportunity not to commit to participation (video link: https://www.youtube.com/watch?v=dC0OJ8Wnbsg). In addition, participants will receive a monthly letter thanking them for their participation and describing the

importance to the trial of continued data collection alongside their monthly incentive for using the electronic adherence monitor. Participants receive incentives: $25 for each completed study visit and $10/month for six months for using the electronic adherence monitor during the trial. The coaching program will be delivered free of charge. Participants randomized to the control arm will have the opportunity to schedule a free coaching session after completing all follow-up assessments. Participants will be given $15 to defray travel costs for each study visit. If a participant has no other means of transportation and lives within 10 miles of the clinic, transportation will be arranged instead of the travel reimbursement.

The research associates will make multiple attempts to re-schedule missed appointments within a one-month window. We will also employ a combination of retention strategies, including pre-appointment reminder calling, phone follow-up of missed appointments, updating of contact information at each visit, and distribution of materials with study phone numbers. Participants will receive a birthday card mailed to their address from the study team.

Intervention and Control Conditions
The SEE Program is a six-month personalized glaucoma coaching program. The glaucoma coach goes over tailored education from a web-based application and uses MI-based counseling to help glaucoma patients identify their barriers to optimal medication adherence and explore potential solutions. Participants attend three in-person coaching sessions. The coach also gives between-session support through four phone calls, with two phone calls between the first and second session and one phone call after each following session. At each session after baseline, the coach uses the medication adherence score to help participants identify barriers and facilitators to ideal medication adherence. Participants can elect to receive any or none of the following alarms/reminders: an audible alarm, a visual alarm, a text message one hour after a dose of medication is missed or a text message one hour after a dose of medication is missed. (**Figure 1**) The control group received control care, receiving three mailings of glaucoma educational materials from leading eye care institutions in parallel to the three in-person coaching sessions. Glaucoma medication adherence will be monitored electronically for all participants between the baseline visit and the exit visit six months later.

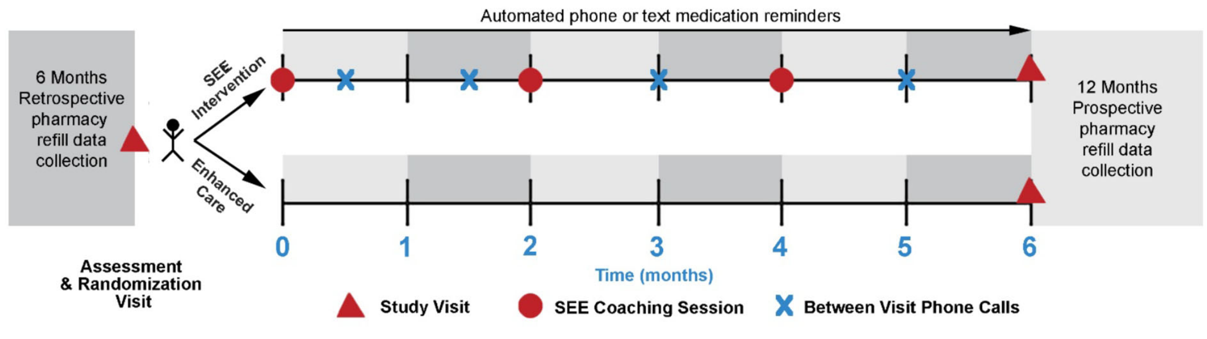

Figure 1. Schedule of SEE Trial visits for Intervention and Control arms; Primary Outcome Assessment at 6 months

Intervention: During the first in-person coaching session, the glaucoma coach uses a web-based application to generate tailored glaucoma educational materials and teach eye drop instillation. The education is tailored on the following variables: name, gender, race/ethnicity, type of glaucoma, glaucoma test results (visual field tests and optic nerve photographs), previous laser or incisional glaucoma surgeries, recommended glaucoma medications, physician's name, cell phone and internet usage, social support, and barriers to adherence. The application also generates MI-based counseling prompts to guide the conversation between the coach and

patient. The coach shows the patient the tailored audio-visual educational materials on a large tablet (9"x12") that can be enlarged as needed for patients who are visually impaired. Text is read aloud. Videos can be played at higher volumes to ensure patients can hear. During the session, the coach helps the patient identify barriers to optimal adherence and uses the person's strengths and motivations to guide potential solutions. Over the course of the counseling session, the coach helps the patient put together a list of questions to ask the doctor at the next visit. At the end of each session, the coach uses the web-application to create a written action plan of the next steps to integrate medication taking into the patient's daily routine. Patients receive adherence monitors and choose any or all of their preferred modalities for receiving reminders when a medication dose is missed: an alarm (light or sound) and/or an automated phone call or text message.
Between-session phone calls are tailored to the patient's current level of adherence as measured by the electronic adherence monitor and focus on problem solving issues that arise. Phone calls are made using Google Voice software allowing the glaucoma coach to use his/her cell phone to call patients from a Google Voice number. This program facilitates a recorded history of call and text conversations. During the second in-person session, patients will discuss their motivation to take care of their vision and what strengths they have that they can use to enact their plan, practice instilling eye drops, and go over their daily routine for eye drop use. In the third session, patients can choose what parts of the personalized glaucoma educational content they would like to review and discuss and trouble shoot any new barriers that may have arisen. (SEE website: https://seeglaucoma.org)

Coaches have two different notes sections in the application. One is to record information for themselves to remember about the participant. The other notes section is for the counselors to record any questions the participants have that would be good to ask their physician. The counselor then prints out a list of questions for the participant to take to their upcoming physician visit.

Patients will receive approximately 160 minutes of counseling (120 minutes in-person and four 10-minute telephone calls). This is within the range of counseling time (106±92.4 minutes) that achieved significant behavior change in a meta-analysis.[19] The glaucoma coaches update the participants' ophthalmologist on their adherence and action plan. Participants can call their coach if questions arise.

Control condition, control care: The purpose of the trial is to measure whether the SEE glaucoma counseling program improves glaucoma medication adherence compared to current practice. Therefore, we chose to use control physician care as the comparator to control for the attention effect given by additional education without greatly changing the comparator group from what would currently be delivered in clinic. The control group will receive non-tailored educational content by mail from current gold standard providers (American Academy of Ophthalmology, National Eye Institute and Glaucoma Research Foundation) in three doses parallel to the three in-person coaching sessions.

OUTCOMES

Primary Outcome: Six-Month Medication Schedule Adherence. Medication adherence will be measured objectively using electronic monitors with a bottle-in-bottle technique where all glaucoma medications are placed inside separate electronic pill bottles. When the bottle cap is removed, the time and date stamp is sent to our database. An adherent event is defined as using an eye drop medication within a specified time window of a dose on the previous day. For example, for an eye drop medication dosed once per day, an adherent event is defined as taking the medication within 24 ± 4 hours of the previous day's dose. A two-hour window is used for twice daily dosed medications and a one-hour and 20 minute window for three times daily medications. We include this time window because the biological efficacy of eye drop medications decline when not taken on time. We will compare the current day's doses to the previous day's corresponding doses, ensuring that opening a bottle multiple times prior to a clinic visit does not inflate the adherence metric. Adherence will be calculated as the proportion of doses taken on time divided by total doses prescribed over the six-month study period. For participants on more than one medication, adherence will be first measured at the medication level and then aggregated to the person level by dividing the total number of doses of all medication(s) taken on time by the total number of doses prescribed. Our primary outcome is the mean adherence in the control and intervention groups. We will also assess the proportion of patients who are ≥80% adherent.

Secondary Outcome: Glaucoma-related Distress. Glaucoma-related distress, or the emotional distress participants experience related to having glaucoma, will be measured using the Diabetes Distress Scale adapted for glaucoma (Cronbach's α= 0.93).[26] The Diabetes Distress Scale consists of 17 items with four subscales that measure emotional burden (5 items), physician-related distress (4 items), regimen-related distress (5 items), and diabetes-related interpersonal distress (3 items).[27] Responses are measured on a 6-point Likert scale from strongly disagree/least

distress (1) to strongly agree/most distress (6). The scale is scored as an overall mean of all question responses (composite score), as well as a mean of questions included only in subscales of emotional burden, physician-related distress, regimen-related distress, and interpersonal distress. The Emotional Burden subscale contains items that describe feeling like the glaucoma is in control of the participant's life and visual fate despite their best efforts, as opposed to the participant feeling like they can control the disease. The Regimen-related distress subscale contains items that describe how the participants feel they are failing with their glaucoma medication routine, and they are not motivated to keep up with it on a daily basis. In our pilot validation study of three items from the regimen-related distress and emotional burden subscales in a glaucoma population, we found a significant association of increased glaucoma-related distress with poor electronically monitored glaucoma medication adherence (adjusted β = -2.47, standard error (SE) = 0.61, *P*=0.0001).[26]

Exploratory Outcome: Intraocular Pressure (IOP). Change in IOP between the baseline and exit visit will be assessed. We will measure IOP in both arms using the iCare (Tiolat Oy, Helsinki, Finland), a method that does not require corneal anesthesia. The iCare has been shown to correlate well with gold standard IOP measurement by Goldmann applanation tonometry.[28] We will obtain three reliable (assessed by iCare) measures of IOP at each study visit and use the median as the study visit IOP which will be recorded both in the REDCap database and in the electronic health record to communicate with the participants' ophthalmologist. We will obtain three measurements due to the variability in IOP measurement. IOP control greatly reduces vision loss from glaucoma and is used as a surrogate marker of glaucoma control in drug and device trials and in clinical practice.[29–31] However, given the short duration of the SEE Program, we would expect this intervention to cause at most a small reduction in IOP from baseline to six months, even if diurnal measures of IOP or obtaining IOP at a set time were feasible, which is not the case. We are not powered to detect small changes in IOP. Assessing whether there is a trend toward lower IOP in intervention participants will inform future larger scale trials with a measure of IOP control as a primary outcome.

STATISTICAL ANALYSIS PLAN

We will perform intent-to-treat analysis of all randomized participants to evaluate our primary, secondary and exploratory outcomes. We will compare the primary outcome variable, mean adherence, between the two groups using ANOVA, blocking on clinic. We will also analyze the proportion of subjects achieving ≥80% adherence between the treatment group and control group, with the Cochran-Mantel-Haenszel Test for equality of proportions. We will use Student's t-test on post-trial, 12-month medication possession ratios to assess longer-term effects of the SEE Program compared to control care.

Additionally, we will use pre-trial medication possession ratios in ANCOVA to investigate whether treatment effectiveness is steady across different levels of pre-trial adherence. To investigate trends in adherence over time, we will calculate adherence monthly. Spaghetti plots will visualize trends within the treatment and control groups (linear, step, decaying effect, etc.). Linear regression will test for trends of adherence over time by treatment group.

For the secondary outcome of glaucoma related distress (GRD), we will score the scale according to the measure's documentation at the baseline and exit visits and calculate change. Descriptive statistics and plots will be generated to understand the distribution of scores and change, overall and stratified by treatment group and clinic. The exit GRD score will be regressed on treatment group, adjusted for baseline GRD and clinic.

For the exploratory IOP outcome, exit IOPs will be analyzed using measures from both eyes. The distribution of IOP and IOP change will be assessed with descriptive statistics and plots. The effect of treatment on IOP will be assessed with a linear mixed regression model, adjusting for baseline IOP and clinic, and controlling for the correlation between eyes of a subject with a random subject effect.

We conducted brief interviews with 25 glaucoma specialists from throughout the US to gauge expert opinion regarding clinically important effect size.[32] Their average recommendation for difference of group proportions of good adherence was 18.5 percentage points (95% CI 15.6-21.5). Their average recommendation for difference of group mean adherence was 17.7 percentage points (95% CI 14.6-20.8). Additionally, mean medication adherence increased by 20 percentage points in our SEE Program pilot study from 65.7% (±10.3) at baseline to 85.7% (±11.8) after the program. With 97 participants in each group, a t-test has 80% power to detect a difference of 8 percentage points in mean adherence between the two groups, if the within group standard deviation (SD) is 20 (0.40 20 = 8). In our preliminary data the SD was 18, but we anticipate and plan for a possibly more diverse study group (calculated using R 'pwr' package). Our glaucoma specialists were divided on the importance of clinically important effect size, so we plan to be adequately powered for both continuous and binary adherence measures. Their average recommendation for difference of group proportions of good adherence was 18.5 percentage points (95% CI 15.6-

21.5). The proposed sample size (n=97 per group) provides 80% power (at two-sided α=0.05) for Pearson's Chi Square Test of Independence to find a difference of 20 percentage points for the proportion of patients attaining good (≥80%) adherence between the intervention and control groups.

For our secondary outcome, with this sample size, we can detect a 1.1-point difference in mean change in Glaucoma Related Distress (scale 6-18) between trial arms if the SD is 2.8, as it was in our preliminary data. For this sample size in our exploratory outcome, we can detect a 1.9 mmHg difference in mean change in IOP between trial arms if the SD is 4.6 mmHg, as it was in our preliminary data. Although we will make concerted efforts to maximize retention, we will prepare for up to a 20% loss to follow-up as was experienced in our preliminary study and will enroll 115 participants in each arm for a total of 230 participants.

DATA AND SAFETY MONITORING PLAN

The study epidemiologist will lead the study team in quarterly review of study recruitment, adverse events, and compliance with the protocol. The study epidemiologist will work with the study statistician to prepare reports of recruitment, retention, and adverse events for the entire team.

We will follow the standard IRBMED AE/ORIO reporting plan.

Intraocular pressure will be measured at the baseline and exit study visits. If the IOP is more than 4mm Hg above the target pressure set by their ophthalmologist or, if no target pressure is set, >21 mmHg, the participant will be walked over to the eye clinic for further evaluation. The research associate will email the participant's ophthalmologist, the sites PI to let them know what transpired and will also send them the note from the study visit and clinic visit through the electronic health record to determine appropriate follow up. If the participant's IOP is above the target but below the threshold for immediate clinical attention, the research associate will email the participant's ophthalmologist, and cc the site PI in addition to sending a notification to the ophthalmologist through the electronic health record to determine the appropriate follow up interval. The research associate will keep a log of all participants whose eye pressure met criteria to be walked to clinic to be reviewed during the quarterly reports.

SECONDARY SITE

University of Michigan will have a contract in place with Henry Ford Health Systems in place prior to beginning any study activities.

DEVIATIONS FROM PLANNED STATISTICAL ANALYSIS

The primary outcome variable, percent medication adherence, was compared between intervention and control groups using Wilcoxon rank-sum tests. This is a deviation from the planned analysis of ANOVA with blocking on clinic due to due to non-normality of the adherence outcome and differences in sample characteristics by site (**Table S5**). These site differences will be incorporated into multivariable models to adjust for differences and investigating moderating effects with treatment on the outcome in future analyses. The proportion of participants achieving ≥80% adherence was analyzed with a Chi-square test rather than a Cochran-Mantel-Haenszel test to again allow for future analysis that will more thoroughly account for investigating site differences on the intervention and adherence. Further, the primary outcome was analyzed using 4 difference calculations of adherence (censored, worst-case, best-case, and worst-best-case scenarios) and therefore *P* values were adjusted for multiplicity using Holm's procedure. For the secondary outcome (GRD), we modeled change in GRD instead of exit GRD as we were more interested in GRD change and its relation to a minimally important difference, as described by Banks J et al.[33] For the exploratory outcome (IOP), we modeled better eye and worse eye IOP instead of IOP of both eyes with adjustment for inter-eye correlation because glaucoma severity is not always bilateral and medications are known to be less robust in the worse eye as described by Bayer A et al.[34] Similar to the primary outcome, the secondary and exploratory outcome analyses were not adjusted for site due to differences in sample characteristics between site that will more thoroughly be investigated in multivariable models to adjust for differences and investigate moderating effects with treatment on these outcomes.

The study protocol and SAP also propose analysis of medication possession ratios from pharmacy refill data, investigation of adherence trends over time, and qualitative assessment of participant interviews regarding study experience. These topics are all outside the scope of the current manuscript and are all planned for future reporting.

REFERENCES

1. Garway-Heath DF, Crabb DP, Bunce C, et al. Latanoprost for open-angle glaucoma (UKGTS): a randomised, multicentre, placebo-controlled trial. *Lancet*. 2015;385(9975):1295-1304.

2. Newman-Casey PA, Blachley T, Lee PP, Heisler M, Farris KB, Stein JD. Patterns of Glaucoma Medication Adherence over Four Years of Follow-Up. *Ophthalmology*. 2015;122(10):2010-2021.

3. Reardon G, Kotak S, Schwartz GF. Objective assessment of compliance and persistence among patients treated for glaucoma and ocular hypertension: a systematic review. *Patient Prefer Adherence*. 2011;5:441-463.

4. *Adherence to Long-Term Therapies: Evidence for Action. Section II – Improving Adherence Rates: Guidance for Countries*. World Health Organization; 2003:17-44. https://iris.who.int/bitstream/handle/10665/42682/9241545992.pdf

5. Murakami Y, Lee BW, Duncan M, et al. Racial and ethnic disparities in adherence to glaucoma follow-up visits in a county hospital population. *Arch Ophthalmol*. 2011;129(7):872-878.

6. Stewart WC, Chorak RP, Hunt HH, Sethuraman G. Factors associated with visual loss in patients with advanced glaucomatous changes in the optic nerve head. *Am J Ophthalmol*. 1993;116(2):176-181.

7. Dreer LE, Girkin CA, Campbell L, Wood A, Gao L, Owsley C. Glaucoma medication adherence among African Americans: program development: Program development. *Optom Vis Sci*. 2013;90(8):883-897.

8. Killeen OJ, MacKenzie C, Heisler M, Resnicow K, Lee PP, Newman-Casey PA. User-centered Design of the eyeGuide: A Tailored Glaucoma Behavior Change Program. *J Glaucoma*. 2016;25(10):815-821.

9. Newman-Casey PA, Niziol LM, Lee PP, Musch DC, Resnicow K, Heisler M. The Impact of the Support, Educate, Empower Personalized Glaucoma Coaching Pilot Study on Glaucoma Medication Adherence. *Ophthalmol Glaucoma*. 2020;3(4):228-237.

10. Hollenhorst CN, Elliott V, Heisler M, Schneider K, Resnicow K, Newman-Casey PA. Patient Experience during the Support, Educate, Empower Glaucoma Coaching Program to Improve Medication Adherence: A Pilot Study. *Ophthalmol Glaucoma*. 2020;3(4):238-252.

11. Newman-Casey PA, Killeen O, Miller S, et al. A Glaucoma-Specific Brief Motivational Interviewing Training Program for Ophthalmology Para-professionals: Assessment of Feasibility and Initial Patient Impact. *Health Commun*. 2020;35(2):233-241.

12. Health promotion glossary. June 16, 1998. Accessed April 7, 2025. https://www.who.int/publications/i/item/WHO-HPR-HEP-98.1

13. Stepanikova I, Cook KS. Effects of poverty and lack of insurance on perceptions of racial and ethnic bias in health care: Effects of poverty and lack of insurance. *Health Serv Res*. 2008;43(3):915-930.

14. Lee C, Ayers SL, Kronenfeld JJ. The association between perceived provider discrimination, healthcare utilization and health status in racial and ethnic minorities. *Ethn Dis*. 2009;19(3):330-337.

15. Hausmann LRM, Gao S, Mor MK, Schaefer JH Jr, Fine MJ. Understanding racial and ethnic differences in patient experiences with outpatient health care in Veterans Affairs Medical Centers. *Med Care*. 2013;51(6):532-539.

16. Tucker CM, Wippold GM, Smith TM, et al. Association of health self-empowerment with health-promoting behaviors among chronically ill African American and non- Hispanic White adolescents. *J Health Care Poor Underserved*. 2014;25(4):2019-2031.

17. Miller WR, Rollnick S. Meeting in the middle: motivational interviewing and self-determination theory. *Int J Behav Nutr Phys Act*. 2012;9(1):25.

18. Rubak S, Sandbaek A, Lauritzen T, Christensen B. Motivational interviewing: a systematic review and meta-analysis. *Br J Gen Pract*. 2005;55(513):305-312.

19. Lundahl B, Moleni T, Burke BL, et al. Motivational interviewing in medical care settings: a systematic review and meta-analysis of randomized controlled trials. *Patient Educ Couns*. 2013;93(2):157-168.

20. Welcome to the Motivational Interviewing Website! MINT. Accessed June 10, 2025. https://motivationalinterviewing.org/

21. Working Group I to the Fifth Assessment Report of the Intergovernmental Panel on Climate Change Stocker, T. F. , D. Qin, G.-K. Plattner, M. Tignor, S. K. Allen, J. Boschung, A. Nauels, Y. Xia. Summary for Policymakers. In: Midgley VBAP, ed. *Climate Change 2013: The Physical Science Basis*. Cambridge University Press; 2013:3-29.

22. McMaster F, Resnicow K. Validation of the one pass measure for motivational interviewing competence. *Patient Educ Couns*. 2015;98(4):499-505.

23. Cho J, Niziol LM, Lee PP, et al. Comparison of Medication Adherence Assessment Tools to Identify Glaucoma Medication Nonadherence. *Ophthalmol Glaucoma*. 2022;5(2):137-145.

24. Yohannan J, Wang J, Brown J, et al. Evidence-based criteria for assessment of visual field reliability. *Ophthalmology*. 2017;124(11):1612-1620.

25. Charlson ME, Pompei P, Ales KL, MacKenzie CR. A new method of classifying prognostic comorbidity in longitudinal studies: development and validation. *J Chronic Dis*. 1987;40(5):373-383.

26. Cho J, Song M, Niziol LM, et al. Patient-Centered Outcomes After a Medication Adherence Intervention: a Pilot Study. *J Glaucoma*. 2023;32(10):891-899.

27. Polonsky WH, Fisher L, Earles J, et al. Assessing psychosocial distress in diabetes: development of the diabetes distress scale. *Diabetes Care*. 2005;28(3):626-631.

28. Nakakura S. Icare® rebound tonometers: review of their characteristics and ease of use. *Clin Ophthalmol*. 2018;12:1245-1253.

29. Gedde SJ, Chen PP, Heuer DK, et al. The Primary Tube Versus Trabeculectomy Study: Methodology of a multicenter randomized clinical trial comparing tube shunt surgery and trabeculectomy with mitomycin C. *Ophthalmology*. 2018;125(5):774-781.

30. Katz LJ, Steinmann WC, Kabir A, Molineaux J, Wizov SS, Marcellino G. Selective laser trabeculoplasty versus medical therapy as initial treatment of glaucoma: A prospective, randomized trial. *J Glaucoma*. 2012;21(7):460-468.

31. Prum BE Jr, Rosenberg LF, Gedde SJ, et al. Primary Open-angle glaucoma preferred practice pattern(®) guidelines. *Ophthalmology*. 2016;123(1):P41-P111.

32. Kolli A, Daniel-Wayman S, Newman-Casey PA. The Minimal Clinically Important Difference in Glaucoma Medication Adherence: Interviews of Glaucoma Experts. *Ophthalmic Res*. 2021;64(3):524-528.

33. Banks J, Amspoker AB, Vaughan EM, Woodard L, Naik AD. Ascertainment of minimal clinically important differences in the Diabetes Distress Scale-17: A secondary analysis of a randomized clinical trial: A secondary analysis of a randomized clinical trial. *JAMA Netw Open*. 2023;6(11):e2342950.

34. Bayer A, Henderer JD, Kwak T, Myers J, Fontanarosa J, Spaeth GL. Clinical predictors of latanoprost treatment effect. *J Glaucoma*. 2005;14(4):260-263.
