# Supplementary material for: Effect of the Support, Educate, Empower Personalized Glaucoma Coaching Program on Medication Adherence: The SEE Program Randomized Clinical Trial
Source: JAMA Ophthalmol. 2026 Feb 26;144(4):299–306. doi: 10.1001/jamaophthalmol.2026.0001 (PMC12947086; doi:10.1001/jamaophthalmol.2026.0001)
Supplement: Supplement 2. — eTable 1. Glaucoma Related Distress Scale eTable 2. Comparison of participant characteristics between those who did and did not complete the SEE study eTable 3. Descriptives for duration of counseling received by participants randomized to the Support, Educate, Empower (SEE) intervention, stratified by those who finished and did not finish the 6-month intervention eTable 4. Summary of glaucoma related distress and intraocular pressure at baseline and exit visits for intervention and control groups eTable 5. Protocol deviations and adverse events eFigure. The distribution of medication adherence over time during the 6 month study period, stratified by SEE counseling intervention and control groups [file jamaophthalmol-e260001-s002.pdf]

Supplemental Online Content

Newman-Casey PA, Niziol, LM, Lu MC, et al. Effect of the support, educate, empower personalized glaucoma coaching program on medication adherence: a randomized clinical trial. *JAMA Ophthalmol*. Published online February 26, 2026. doi:10.1001/jamaophthalmol.2026.0001

eTable 1. Glaucoma Related Distress Scale

eTable 2. Comparison of participant characteristics between those who did and did not complete the SEE study

eTable 3. Descriptives for duration of counseling received by participants randomized to the Support, Educate, Empower (SEE) intervention, stratified by those who finished and did not finish the 6-month intervention

eTable 4. Summary of glaucoma related distress and intraocular pressure at baseline and exit visits for intervention and control groups

eTable 5. Protocol deviations and adverse events

eFigure. The distribution of medication adherence over time during the 6 month study period, stratified by SEE counseling intervention and control groups

This supplemental material has been provided by the authors to give readers additional information about their work.

© 2026 Newman-Casey PA et al. *JAMA Ophthalmology*.

eTable1. Glaucoma Related Distress Scale

Responses to each item are assessed on a six-point Likert scale from strongly disagree (1, least distress) to disagree (2), slightly disagree (3), slightly agree (4), agree (5), and strongly agree (6, most distress). The subscales are presented below with corresponding item numbers.

Emotional Burden

1. Feeling that glaucoma is taking up too much of my mental and physical energy every day.

3. Feeling angry, scared, and/or depressed when I think about living with glaucoma

8. Feeling that glaucoma controls my life.

11. Feeling that I will end up with serious long-term vision loss, no matter what I do.

14. Feeling overwhelmed by the demands of living with glaucoma.

Regimen related distress

5. Feeling that I am not seeing my eye doctor to have my eye pressure checked frequently enough.

6. Feeling that I am often failing with my glaucoma regimen

10. Not feeling confident in my day-to-day ability to manage glaucoma.

12. Feeling that I am not sticking closely enough to my eye drop medication schedule.

16. Not feeling motivated to keep up my glaucoma self-management

Physician Related Distress

2. Feeling that my doctor doesn’t know enough about glaucoma and glaucoma care.

4. Feeling that my doctor doesn’t give me clear enough directions on how to manage my glaucoma.

9. Feeling that my doctor doesn’t take my concerns seriously enough.

15. Feeling that I don’t have a doctor who I can see regularly about my glaucoma.

Interpersonal Distress

7. Feeling that friends or family are not supportive enough of my selfcare efforts (e.g., taking my eyedrops on time).

13. Feeling that friends or family don’t appreciate how difficult living with glaucoma can be

17. Feeling that friends or family don’t give me the emotional support that I would like.

© 2026 Newman-Casey PA et al. *JAMA Ophthalmology*.

eTable2. Comparison of participant characteristics between those who did and did not complete the SEE study.

	Incomplete (n=38)			Complete (n=197)			Difference		
**Continuous Variable**	N	Mean (SD)	Median	N	Mean (SD)	Median	Mean (95% CI)	*P* value*	Adj *P* Value
Age (years)	38	65.1 (11.3)	66.1	197	67.7 (10.8)	68.7	-2.6 (-6.4,1.2)	.20	1.00
Self-reported Adherence (%)	38	63.7 (17.5)	70.0	197	63.9 (18.1)	70.0	-0.2 (-6.4,6.1)	.90	1.00
Better eye MD (dB)	35	-4.90 (5.98)	-3.73	192	-4.91 (7.46)	-2.1	0.004 (-2.63,2.63)	.50	1.00
Worse eye MD (dB)	35	-11.89 (9.24)	-11.36	192	-9.99 (9.54)	-6.2	-1.90 (-5.34,1.54)	.16	1.00
Better eye IOP (mmHg)	34	16.6 (7.3)	17.0	191	16.0 (6.7)	15.0	0.6 (-1.9,3.1)	.52	1.00
Worse eye IOP (mmHg)	35	14.5 (4.3)	14.0	192	15.6 (6.1)	15.0	-1.0 (-3.2,1.1)	.51	1.00
Better eye logMAR VA	34	0.76 (2.05)	0.14	192	0.40 (1.21)	0.10	0.37 (-0.37,1.10)	.12	1.00
Snellen Equivalent		20/115	20/28		20/50	20/25	3.7 lines		
Worse eye logMAR VA	34	0.39 (0.66)	0.26	192	0.28 (0.70)	0.10	0.11 (-0.15,0.37)	.04	.82
Snellen Equivalent		20/49	20/36		20/38	20/25	1.1 lines		
Number Baseline Daily Doses	37	3.3 (2.1)	3.0	197	3.1 (2.0)	3.0	0.2 (-0.5,0.9)	.61	1.00
CCI	38	5.5 (3.6)	5.0	197	5.5 (3.5)	5.0	-0.07 (3.5,0.63)	.93	1.00
Glaucoma-related Distress	38	2.7 (0.8)	2.6	197	2.5 (0.8)	2.4	0.26 (-0.03, 0.56)	.08	1.00
**Categorical Variables**	**Frequency (%)**			**Frequency (%)**			**% (95% CI)**	***P* value****	**Adj *P* Value**

© 2026 Newman-Casey PA et al. *JAMA Ophthalmology*.

Site					
UM	20 (52.6)	87 (44.2)	8.5 (-8.9, 25.8)	.34	1.00
HFHS	18 (47.4)	110 (55.8)	-8.5 (-25.8, 8.9)		
Number of Baseline Medications					
1	10 (27.0)	79 (40.1)	-13.1 (-28.9, 2.8)	.02	.50
2	20 (54.1)	65 (33.0)	21.1 (3.7, 38.4)		
3	4 (10.8)	42 (21.3)	-10.5 (-22.0, 1.0)		
4	2 (5.4)	11 (5.6)	-0.2 (-8.1, 7.8)		
5	1 (2.7)	0 (0.0)	2.7 (-2.5, 7.9)		
Sex - Male	17 (44.7)	93 (47.4)	-2.7 (-20.0, 14.6)	.76	1.00
Ethnicity - Hispanic	1 (2.9)	5 (2.9)	0.001 (-6.1, 6.3)	1.00	1.00
Race					
Black	20 (52.6)	119 (63.0)	-10.3 (-27.6, 7.0)	.10	1.00
White	11 (29.0)	57 (30.2)	-1.2 (-17.1, 14.6)		
Asian	3 (7.9)	7 (3.7)	4.2 (-4.8, 13.2)		
Other	4 (10.5)	6 (3.2)	7.4 (-2.7, 17.4)		
Education					
Less than High School	2 (5.4)	9 (4.7)	0.7 (-7.2, 8.6)	.08	1.00

© 2026 Newman-Casey PA et al. *JAMA Ophthalmology*.

High School Diploma	12 (32.4)	31 (16.2)	16.3 (0.3, 32.2)		
Some College	5 (13.5)	60 (31.3)	-17.7 (-30.6, 4.9)		
College Degree	8 (21.6)	42 (21.9)	-0.003 (-14.8, 14.2)		
Graduate Degree	10 (27.0)	50 (26.0)	1.0 (-14.6, 16.6)		
Income					
Less than $20,000	10 (27.8)	26 (15.5)	12.3 (-3.3, 27.9)		
$20,000 to $40,000	9 (25.0)	45 (26.8)	-1.8 (-17.4, 13.9)		
$41,000 to $60,000	3 (8.3)	35 (20.8)	-12.5 (-23.4, -1.6)		
$61,000 to $80,000	3 (8.3)	16 (9.5)	-1.2 (-11.3, 8.9)	.23	1.00
$81,000 to $100,000	5 (13.9)	11 (6.6)	7.3 (-4.6, 19.2)		
$101,000 to $120,000	1 (2.8)	13 (7.7)	-5.0 (-11.7, 1.8)		
Greater than $120,000	5 (13.9)	22 (13.1)	0.8 (-11.6, 13.2)		
Health Insurance	37 (97.4)	194 (99.5)	-2.1 (-7.3, 3.1)	.30	1.00
Prescription Coverage	33 (89.2)	181 (93.8)	-4.6 (-15.2, 6.0)	.30	1.00

SEE, Support, Educate, Empower; SD, Standard Deviation; Adj, Adjusted for multiple comparisons using Holm's method; MD, Mean Deviation; dB, Decibel; IOP, Intraocular Pressure; mmHg, millimeters of Mercury; logMAR, logarithm of Minimum Angle of Resolution; VA, Visual Acuity; CCI, Charlson Comorbidity Index; UM, University of Michigan; HFHS, Henry Ford Health System; Note: better and worse eye categorizations were determined by baseline MD; *2-sample t-test (age) or Wilcoxon rank-sum tests (all other continuous measures); **Chi-square test or Fisher's exact test *when cell counts <5)

© 2026 Newman-Casey PA et al. *JAMA Ophthalmology*.

eTable3. Descriptives for duration of counseling received by participants randomized to the Support, Educate, Empower (SEE) intervention, stratified by intervention sessions and phone calls received.

			Duration (minutes)	
**Interventions Received**	n	$n_{non\text{-}missing}$	Mean (SD)	Median
Sessions				
0	4	4	0 (--)	0.0
1	12	11	72.7 (19.6)	70.0
2	8	8	109.0 (25.3)	114.5
3	93	81	134.6 (36.6)	142.0
Phone Calls				
0	13	13	0 (--)	0.0
1	18	17	12.8 (5.2)	12.0
2	31	23	22.8 (9.7)	22.0
3	30	23	35.9 (9.9)	34.8
4	25	24	40.4 (14.3)	38.0
Total	117	90	143.1 (56.1)	154.5

SD, Standard Deviation; note: duration statistics only calculated on the sample with non-missing duration

© 2026 Newman-Casey PA et al. *JAMA Ophthalmology*.

eTable4. Summary of glaucoma related distress and intraocular pressure at baseline and exit visits for intervention and control groups[1].

	SEE Intervention (n=117)		Control (n=118)	
**Glaucoma Related Distress**	n	Mean (SD), Median	n	Mean (SD), Median
Baseline Visit				
Composite	90	2.5 (0.9), 2.4	107	2.4 (0.8), 2.4
Emotional Burden	90	2.7 (1.2), 2.4	107	2.5 (1.0), 2.4
Physician-related Distress	90	1.9 (1.0), 1.8	107	1.8 (0.9), 1.5
Regimen-related Distress	90	3.0 (1.2), 3.0	107	2.9 (1.1), 3.0
Interpersonal Distress	90	2.2 (1.2), 2.0	107	2.3 (1.1), 2.0
Exit Visit				
Composite	90	1.9 (0.8), 1.9	107	2.2 (0.9), 2.0
Emotional Burden	90	2.1 (1.1), 2.0	107	2.4 (1.2), 2.0
Physician-related Distress	90	1.7 (0.9), 1.5	107	1.8 (1.0), 1.5
Regimen-related Distress	90	1.9 (0.8), 1.9	107	2.4 (1.0), 2.2
Interpersonal Distress	90	1.9 (1.0), 1.7	107	2.0 (1.2), 2.0
Change in Glaucoma Related Distress				
Composite	90	-0.6 (0.9), -0.6	107	-0.2 (0.7), -0.2
Emotional Burden	90	-0.5 (1.0), -0.2	107	-0.1 (1.0), -0.2
Physician-related Distress	90	-0.2 (1.0), 0.0	107	-0.0 (0.9), 0.0
Regimen-related Distress	90	-1.1 (1.2), -1.2	107	-0.5 (1.0), -0.4
Interpersonal Distress	90	-0.3 (1.2), 0.0	107	-0.2 (1.1), 0.0
**Intraocular Pressure**	n	Mean (SD), Median	n	Mean (SD), Median
Baseline Visit				
Median IOPs - Worse Eye	87	15.52 (5.74), 15.00	102	15.23 (5.33), 14.50
Median IOPs - Better Eye	88	15.93 (6.93), 15.00	102	14.86 (4.33), 14.00
Exit Visit				
Median IOPs - Worse Eye	87	15.41 (6.05), 14.00	102	15.11 (5.44), 14.00
Median IOPs - Better Eye	88	14.82 (4.59), 14.00	102	14.72 (4.48), 14.00

© 2026 Newman-Casey PA et al. *JAMA Ophthalmology*.

Intraocular Pressure	SEE Intervention (n=117) n	Mean (SD), Median	Control (n=118) n	Mean (SD), Median
Change in IOP				
Median IOPs - Worse Eye	87	-0.10 (5.88), 0.00	102	-0.12 (5.18), 0.00
Median IOPs - Better Eye	88	-1.11 (5.09), -1.00	102	-0.14 (3.69), 0.00

SEE, Support, Educate, Empower; SD, Standard Deviation; IOP, Intraocular Pressure; mmHg, millimeters of Mercury; Note: better and worse eye categorizations were determined by baseline MD. [1]Total sample size presented in this table includes participants with IOP values and glaucoma related distress values both at baseline and at the exit visit.

© 2026 Newman-Casey PA et al. *JAMA Ophthalmology*.

eTable5. Protocol deviations and adverse events

Type	Overall (n=235) # (%)	SEE Intervention (n=117) # (%)	Control (n=118) # (%)	*P* value*
**Adverse Event**	26 (11.1)	18 (15.4)	8 (6.8)	0.04
High IOP	25 (10.6)	18 (15.4)	7 (5.9)	0.02
Death	2 (0.9)	0 (0.0)	2 (1.7)	0.50
**Protocol Deviation**	6 (2.6)	4 (3.4)	2 (1.7)	0.45
Informed Consent Process deviation	2 (0.9)	1 (0.9)	1 (0.9)	1.00
Inadvertent/Accidental protocol departure	1 (0.4)	0 (0.0)	1 (0.9)	1.00
Schedule Deviation	3 (1.3)	3 (2.6)	0 (0.)	0.12

*Chi-square or Fisher's Exact test (when cell sizes are <5); Note: One participant had both a high IOP and died during the course of study

© 2026 Newman-Casey PA et al. *JAMA Ophthalmology*.

eFigure1. The distribution of medication adherence over time during the 6 month study period, stratified by Support, Educate, Empower (SEE) counseling intervention and control groups.

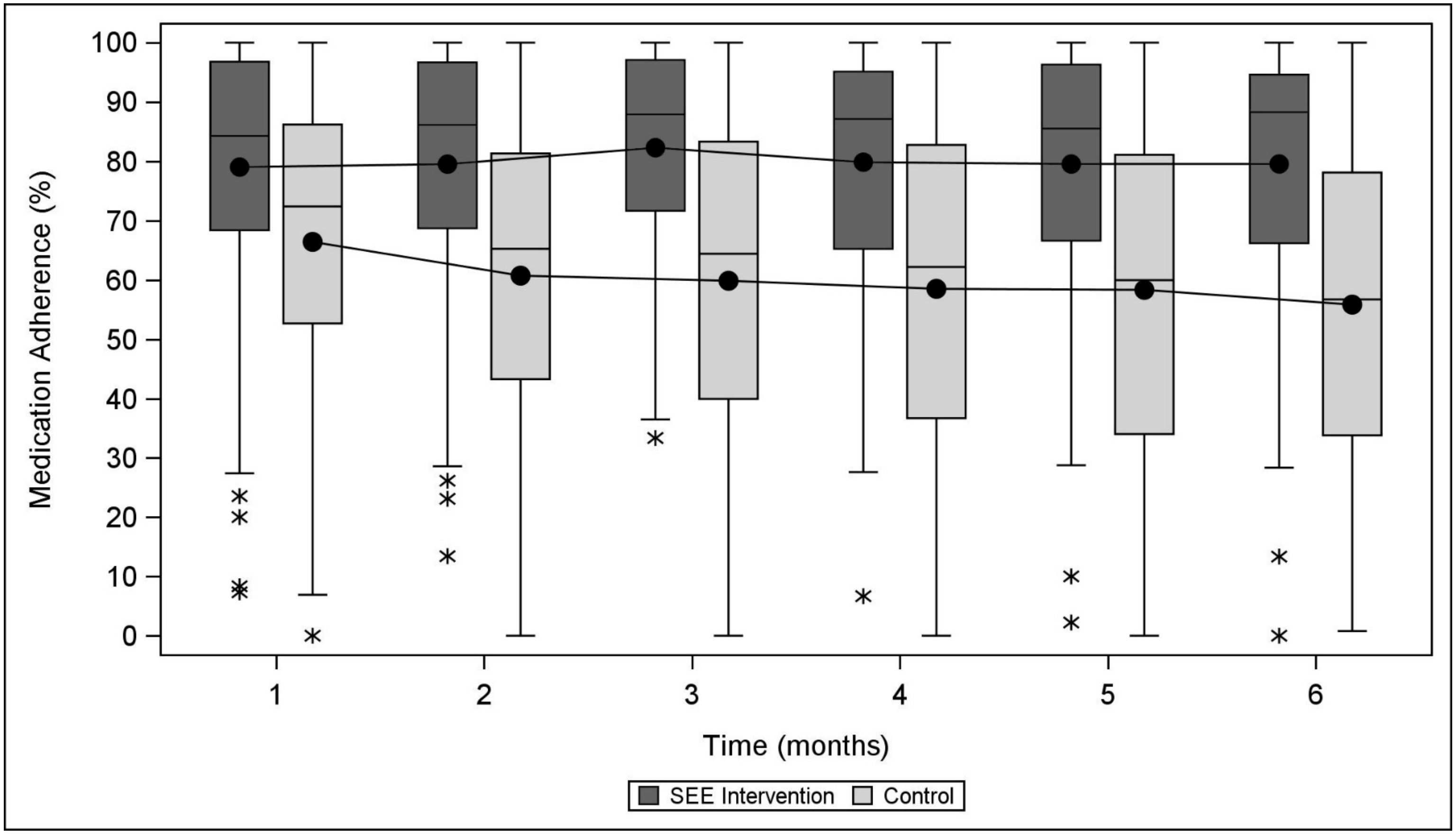

Boxplots display the mean (black dot), median (horizontal bar within the box), interquartile range (IQR, width of the box from the 25th to 75th percentiles), lower fence (-1.5xIQR), upper fence (+1.5xIQR), outliers (values outside ±1.5xIQR).

For the participants in the SEE intervention group, medication adherence during month 1 of the study was not different from medication adherence during month 6 of the study. For participants in the control group, medication adherence during month 1 of study was higher than mediation adherence during month 6 of the study.

© 2026 Newman-Casey PA et al. *JAMA Ophthalmology*.
