# Supplementary material for: Effect of the Support, Educate, Empower Personalized Glaucoma Coaching Program on Medication Adherence: The SEE Program Randomized Clinical Trial
Source: JAMA Ophthalmol. 2026 Feb 26;144(4):299–306. doi: 10.1001/jamaophthalmol.2026.0001 (PMC12947086; doi:10.1001/jamaophthalmol.2026.0001)
Supplement: Supplement 3. — Data sharing statement [file jamaophthalmol-e260001-s003.pdf]

Data Sharing Statement

Newman-Casey. Effect of the Support, Educate, Empower Personalized Glaucoma Coaching Program on Medication Adherence. *JAMA Ophthalmol*. Published February 26, 2026. doi:10.1001/jamaophthalmol.2026.0001

Data
Additional Information: clinicaltrials.gov; https://clinicaltrials.gov/study/NCT04735653?cond=NCT04735653&rank=1; NCT04735653
Data available: Yes
Data types: Other (please specify)
Additional Information: Deidentified participant data will be available on reasonable request and data use agreement.
How to access data: Please email panewman@med.ummich.edu.
When available: With publication

Supporting Documents
Document types: None

Additional Information
Who can access the data: Deidentified participant data will be available on reasonable request for research with a data use agreement.
Types of analyses: Deidentified participant data will be available on reasonable request for research with a data use agreement.
Mechanisms of data availability: With a signed data use agreement.
